# Intraoperative effectiveness of crystalloid and colloid volume substitution in patients undergoing elective major urological surgery by maintenance of the cardiac index within normal range

**DOI:** 10.1186/cc9505

**Published:** 2011-03-11

**Authors:** P Szturz, R Kula, J Maca, J Tichy, J Jahoda

**Affiliations:** 1 Faculty Hospital Ostrava, Ostrava, Czech Republic

## Introduction

We compared intraoperative volume effectiveness of crystalloid and colloid substitution aimed to maintain the cardiac index (CI) within the normal range measured by transesophageal Doppler ultrasonography (TED) [1]. We also evaluated the frequency of postoperative complications, length of in-hospital stay and postoperative in-hospital mortality.

## Methods

One hundred and fifteen urological patients were enrolled into the prospective observational clinical study and then randomized into two groups. The first group was treated by volumotherapy based on crystalloids (Cry) *n *= 57, and the second group with colloids (Col) *n *= 58. High-risk surgery criteria were fulfilled in 47% patients in the Cry group and 45% in the Col group. Each patient obtained an esophageal Doppler probe (Hemosonic™100^®^; Arrow International, USA) after induction to general anesthesia and then hemodynamic optimalization (fluid therapy with Ringer's solution or HES 6% 130/0.4 and administration of vasoactive drugs) was started according to TED variables to keep the CI between 2.6 and 3.8 l/min/m^2^. The supplementation of immeasurable fluid losses in the Col group was provided by infusion of Ringer's solution 0.05 ml/kg/minute.

## Results

We observed high initial incidence of CI <2.6 l/min/m^2 ^after induction of general anesthesia (75%) in both groups. There were no significant differences in demographic characteristics, ASA classification, length of surgical procedure, estimated blood loss and CI during surgery. To maintain the CI we used significantly different amounts of crystalloids compared with colloids: means 5,182 ml versus 1,692 ml, respectively. The number of administered blood units was also higher in the Cry group versus the Col group: RBC 52 versus 19, *P *= 0.018, FFP 55 versus 16, *P *= 0.006, respectively. There was more GIT dysfunction in group Cry 31.6% versus 15.5% in the Col group, *P *= 0.05. The number of complications during 28 days on the ICU, overall in-hospital stay and mortality were not statistically significant.

## Conclusions

Crystalloids and colloids are effective in correction of intraoperative flow-related perfusion abnormalities. Different amounts of used crystalloids and colloids proved their unequal pharmacological characteristics (that is, distribution between compartments). The high amount of used transfusion units and postoperative incidence of GIT dysfunction in the Cry group suggests possibly more adverse effects of crystalloids in the perioperative period.
